# Successful reduction of hospital-acquired methicillin-resistant *Staphylococcus aureus* in a urology ward: a 10-year study

**DOI:** 10.1186/1471-2490-13-35

**Published:** 2013-07-18

**Authors:** Manabu Tatokoro, Kazunori Kihara, Hitoshi Masuda, Masaya Ito, Soichiro Yoshida, Toshiki Kijima, Minato Yokoyama, Kazutaka Saito, Fumitaka Koga, Satoru Kawakami, Yasuhisa Fujii

**Affiliations:** 1 Department of Urology, Tokyo Medical and Dental University Graduate School, 1-5-45, Yushima, Bunkyo, Tokyo 113-8519, Japan

**Keywords:** Antibiotic prophylaxis, Hospital infections, Infection control, Methicillin-resistant *Staphylococcus aureus*, Minimally invasive surgery

## Abstract

**Background:**

To eradicate hospital-acquired methicillin-resistant *Staphylococcus aureus* (MRSA) using a stepwise infection control strategy that includes an avoidance of antimicrobial prophylaxis (AMP) based on surgical wound classification and an improvement in operative procedures in gasless single-port urologic surgery.

**Methods:**

The study was conducted at an 801-bed university hospital. Since 2001, in the urology ward, we have introduced the stepwise infection control strategy. In 2007, surveillance cultures for MRSA in all urological patients were commenced. The annual incidence of MRSA was calculated as a total number of newly identified MRSA cases per 1,000 patient days. Trend analysis was performed using a Poisson regression.

**Results:**

Over the study period, 139,866 patients, including 10,201 urology patients, were admitted to our hospital. Of these patients, 3,719 patients, including 134 ones in the urology ward, were diagnosed with MRSA throughout the entire hospital. Although the incidence of MRSA increased throughout the entire hospital (*p* = 0.002), it decreased significantly in the urology ward (*p* < 0.0001). Of the 134 cases, 45 (33.6%) were classified as “imported,” and 89 (66.4%) as “acquired.” In the urology ward, the incidence of acquired MRSA decreased significantly over time (*p* < 0.0001), whereas the incidence of imported MRSA did not change over time (*p* = 0.66). A significant decrease (*p* < 0.0001) in the incidence of clinically significant MRSA infection over time was found.

**Conclusions:**

Stepwise infection control strategy that includes a reduction or avoidance of antimicrobial prophylaxis in minimally invasive surgery can contribute to a reduction in hospital-acquired MRSA.

**Trial registration:**

Current study has approved by the institutional ethical review board (No.1141).

## Background

Methicillin-resistant *Staphylococcus aureus* (MRSA) results in longer hospitalization, increased expenses, and poorer patient prognosis [1]. MRSA has been swiftly increasing worldwide over the past several decades [2]. The indiscriminate use of antibiotics has been identified as an important factor in the increasing dissemination of MRSA [3]. A relationship between the consumption of antibiotics and antimicrobial resistance has been widely documented, leading to the implementation of antimicrobial control policies [4,5]. Although most hospitals have programs to control the use of antibiotics, MRSA has continued to increase in the majority of hospitals [6].

Antimicrobial prophylaxis (AMP) for surgery has been empirically performed to prevent surgical site infection (SSI) but inadequate use of AMP has been pointed out as the cause of the dissemination of MRSA. [3,7] Nowadays, it is widely accepted that a reduction in AMP should be promoted unless it increases the risk of infectious complications in patients. Recently, it was reported that the introduction of minimally invasive surgery can reduce or avoid AMP without increasing the risk of SSI [8]. We have already reported that AMP can be gradually reduced or avoided in gasless single-port urologic surgery [9,10] without increasing the risk of SSI [11-14]. However, it has not yet been determined whether an infection control strategy that includes a reduction or avoidance of AMP in minimally invasive surgery actually reduces the incidence of MRSA.

Japan is known to have the world’s highest prevalence of MRSA among the various strains of *S. aureus*[15]. The incidence of MRSA in our hospital was also relatively high throughout all of the wards in 2000. In order to eradicate MRSA in our urology ward, since 2001 we have implemented a stepwise infection control strategy, including the introduction and development of gasless single-port surgery concomitant with a reduction or avoidance of AMP based on surgical wound classification. Herein, we present our 10-year study, which has successfully reduced the nosocomial transmission of MRSA in our urology ward.

## Methods

### Setting

The study was conducted at Tokyo Medical and Dental University (TMDU) Hospital, which is an 801-bed tertiary-care university hospital with 19 wards, located in central Tokyo, Japan.

In accordance with the Centers for Disease Control and Prevention (CDC) guidelines [16], we have introduced a stepwise infection control strategy into daily clinical practice since 2001. After receiving approval by the institutional review board, we analyzed impact of the strategy on the incidence of MRSA retrospectively. All consecutive patients admitted between January 2000 and December 2010 constituted the study population.

**Figure 1:**
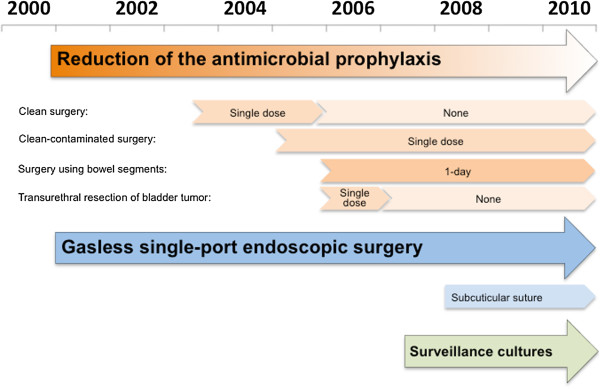
Over view of the stepwise infection control strategy in the urology ward.

After investigating the prevalence of MRSA in 2000, we introduced a stepwise infection control strategy in the urology ward since 2001 (Figure 1), in addition to the following hospital-wide standard precautions for infection control measures. The hospital-wide measures include hand hygiene compliance, strict application of barrier precautions for patients with MRSA, shortened hospital stays, and the continuous education of health-care workers on appropriate hygiene procedures. Patients who were MRSA-colonized on the basis of clinical cultures were placed in contact isolation, and decolonization was not attempted unless necessary.

### Definitions

On admission, we evaluated whether patients were at risk for carrying MRSA. These included patients who were transferred from other hospitals, intensive care units, or nursing homes and patients who were previously colonized with MRSA. An MRSA case was defined as a patient from whom MRSA was recovered from any site, including a patient infected or colonized with MRSA. Each MRSA case was counted only once. MRSA was classified as *imported* when it was identified within 72 hours of admission or if the patient was previously known to be an MRSA carrier; otherwise, it was classified as *acquired in our hospital*[17-19]. Clinically significant MRSA infection (CSMI) was defined as an MRSA infection associated with clinical symptoms and signs of infection that was confirmed by the microbiological demonstration of MRSA [20].

### Stepwise infection control strategy

#### Avoidance and reduction of AMP

The need for AMP generally depends on the surgical wound classification according to the CDC guidelines [16]. Based on the CDC guidelines and several urology guidelines [21,22], we have gradually reduced AMP in urological surgeries for patients without infectious risk. In the pre-intervention period, patients routinely received intravenous second-generation cephalosporin for 3 to 5 days, followed by oral antibiotics for several days. Since April 2001, we have reduced the AMP step by step based on the surgical wound classification [16]. In clean and clean-contaminated surgeries, patients received second-generation cephalosporin administered postoperatively for 2 to 3 days and immediately before the start of the operation. In clean urological surgeries, patients without infectious risk received AMP as follows: between August 2003 and August 2004: ampicillin sodium/sulbactam sodium immediately before the surgery; between September 2004 and September 2005: levofloxacin (LVFX) orally once immediately before the surgery; after October 2005: no AMP was administered [11]. After we confirmed that perioperative infection had not increased, we extended the range of application of reducing AMP. In clean-contaminated urological surgeries, patients received AMP as follows: between January 2005 and September 2005: tazobactam sodium/piperacillin sodium (TAZ/PIPC) intravenously immediately before the surgery and for 3 days afterwards; between October 2005 and September 2006: TAZ/PIPC once immediately before the surgery; after October 2006: LVFX orally once immediately before the surgery [12,13]. In the transurethral resection of bladder tumors, patients received AMP as follows: between April and September 2006: LVFX orally once immediately before the surgery; after October 2006: no AMP was administered [23]. In contaminated urological surgeries using bowel segments, patients received cefmetazole on the operative day only, although prolonged operation and other morbidity risk factors may support the use of a prolonged regimen, which should be within three days according to the European Association of Urology guideline [21]. If a perioperative infection was found, antibiotics were administered immediately according to the drug sensitivity profile of cultured pathogens.

Other than that, in the treatment of urinary tract infection, we use broad-spectrum antimicrobial agents as initial empiric therapy with the intent of covering multiple possible pathogens until microbiological results become available. Once the etiologic pathogen and antimicrobial susceptibility data are available, we use an antibiotic with the narrowest possible spectrum.

#### Improvement of operative procedures and perioperative management

We have developed and performed gasless single-port endoscopic surgery as a novel minimally invasive urologic surgery, which has been covered by the Japanese universal health insurance system since 2008 [9,10]. Through the single port that narrowly permits extraction of the specimen, a wide working space is made by separating the anatomical plane extraperitoneally. This space is maintained with special retractors instead of gas insufflation. The single port is protected by the retractor and the operative field concomitant with the subcutaneous space is rinsed with sterile saline. Since April 2008, skin has been closed by subcuticular suture without additional dressing. After introducing the suture, daily bedside treatment including change of the dressing, which is associated with the nosocomial transmission of MRSA, has been no longer required.

#### Surveillance cultures

Until March 2007, selective screening, mainly urine culture, for MRSA was performed in patients at risk for carrying MRSA at hospital admission and at the time of bedside treatment. In April 2007, we initiated preoperative surveillance cultures of surgical sites, mainly urine cultures, in all patients undergoing surgery to evaluate their infectious risk.

### Microbiological procedures

All of the isolates of MRSA were collected from clinical samples at the clinical microbiology laboratory in our hospital. *S. aureus* was confirmed by Clinical and Laboratory Standards Institute (CLSI) methods [24].

### Statistical analysis

The annual incidence of MRSA is calculated as a total number of newly identified MRSA cases per 1,000 patient days that is the sum of inpatient days of all patients during a year.

Given that MRSA cases are rare compared with the number of admissions, we used Poisson regression model to obtain temporal trends in the incidence of MRSA between 2000 and 2010 [25]. In the model the number of MRSA cases per year was the dependent variable, the actual year of diagnosis was the independent variable, and the logarithm of patient days was an offset [26,27].

The model was log (Number of MRSA cases) = log(Patient days) + B_0_ + B_1_ × Year. B_0_ is the overall intercept and B_1_ is the coefficient for Year.

If the estimation of the coefficient for Year is significantly positive, that indicates the incidence of MRSA significantly increases with time. All statistical analyses were carried out using JMP version 9 (SAS Institute Inc., Cary, NC, USA).

## Results

**Figure 2:**
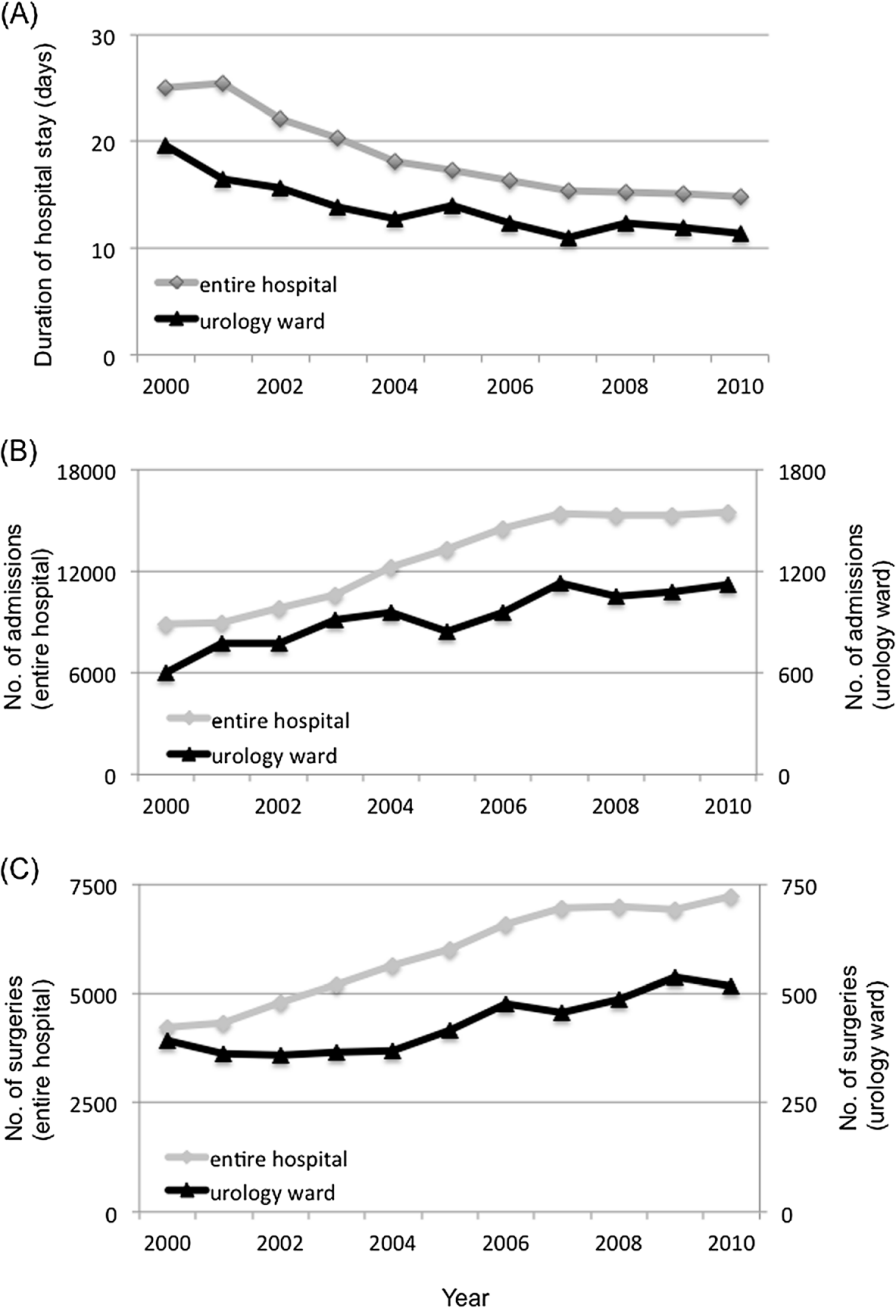
**The annual mean duration of hospital stay, number of admissions and number of surgeries, 2000-2010. (A)** Annual mean duration of hospital stay. **(B)** Annual number of admissions. **(C)** Annual number of surgeries. For both the entire hospital and the urology ward, the mean duration of hospital stay decreased by approximately 40% **(A)**, the annual number of hospital admissions doubled **(B)** and the annual number of surgeries increased by approximately 50% **(C)** over time.

Over the study period, 139,866 patients, including 10,201 urology patients, were admitted to TMDU Hospital. For both the entire hospital and the urology ward, the mean duration of hospital stay decreased by approximately 40% (Figure 2A), the annual number of hospital admissions doubled (Figure 2B) and the annual number of surgeries increased by approximately 50% (Figure 2C) over time. In fact, number of patient days remained unchanged.

**Figure 3:**
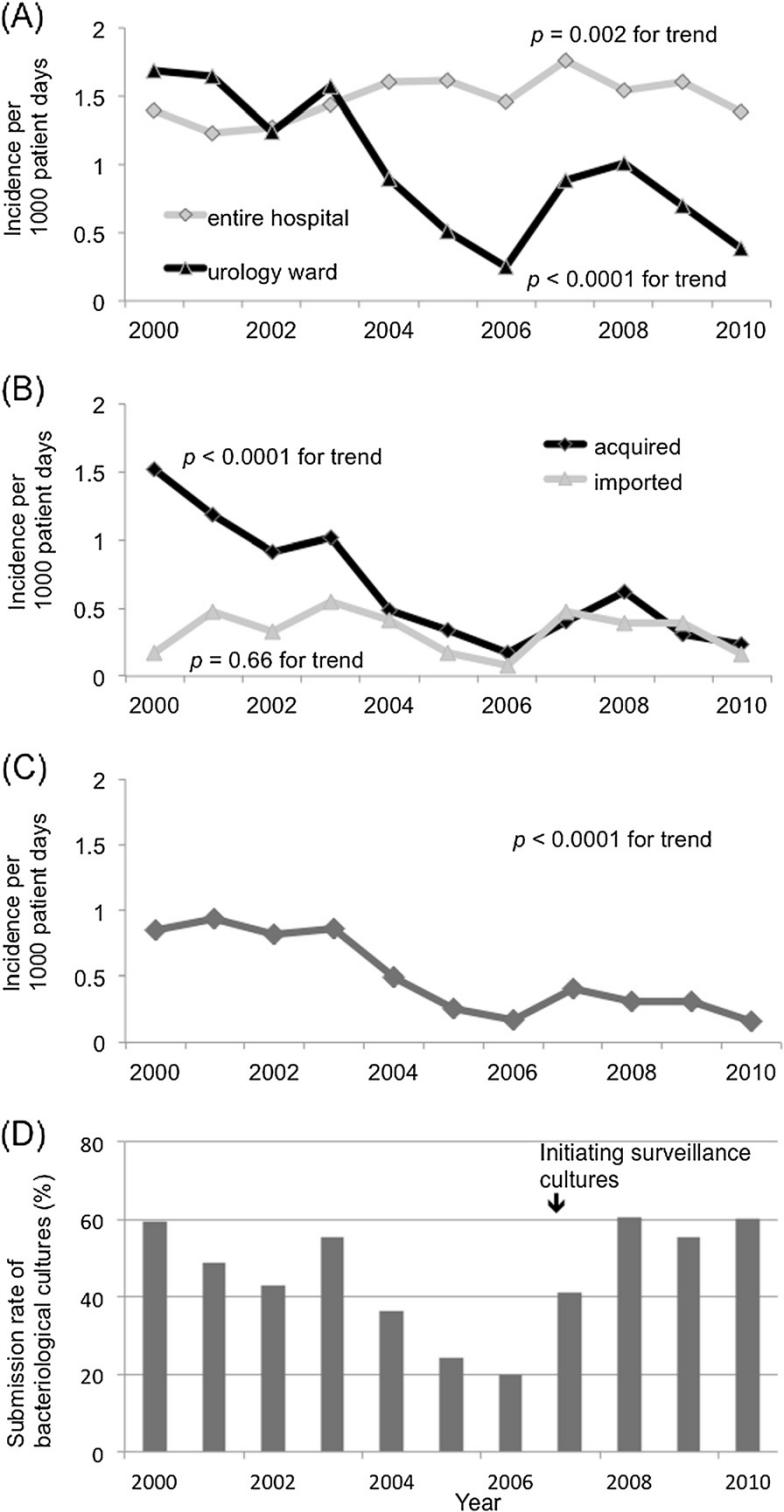
**The annual incidence of methicillin-resistant** ***Staphylococcus aureus*** **per 1,000 patient days and the submission rate of bacteriological cultures in the urology ward, 2000-2010. (A)** Annual incidence of MRSA colonization or infection per 1,000 patient days. **(B)** Annual incidence of MRSA colonization or infection per 1,000 patient days in the urology ward. **(C)** Annual incidence of clinically significant MRSA infection per 1,000 patient days in the urology ward. **(D)** Annual submission rate of bacteriological culture in the urology ward. Although the incidence of MRSA increased throughout the entire hospital, it decreased significantly in the urology ward **(A)**. In the urology ward, the incidence of acquired MRSA decreased significantly, whereas the incidence of imported MRSA did not change over time **(B)**. A significant decrease in the incidence of clinically significant MRSA infection over time was found **(C)**. After introducing surveillance cultures in 2007, the submission rate of bacteriological cultures increased in 2008 **(D)**.

Of these patients, 3,719, including 134 urology patients, were infected or colonized with MRSA. Although the incidence of MRSA increased throughout the entire hospital (coefficient for Year, 0.016; 95% CI, 0.0062 to 0.027; *p* = 0.002), it decreased significantly in the urology ward (coefficient for Year, -0.12; 95% CI, -0.18 to −0.068; *p* < 0.0001) (Figure 3A). Of the 134 cases, 45 (33.6%) were classified as imported, and 89 (66.4%) as acquired in our hospital (Table 1). In the urology ward, the incidence of acquired MRSA decreased significantly over time (coefficient for Year, -0.18; 95% CI, -0.25 to −0.11; *p* < 0.0001), whereas imported MRSA did not change over time (coefficient for Year, -0.020; 95% CI, -0.11 to 0.072; *p* = 0.66) (Figure 3B). Of the 89 acquired MRSA, 54 (61%) had surgical procedures and 66 (74%) received antibiotics before diagnosis of MRSA (Table 1).

**Table 1:** Characteristics of 134 MRSA patients in the urology ward

**Category**		**N=134**	**(%)**
**Gender**	Male	112	84
	Female	22	16
**Mean age (years, range)**		70 (18–94)	
	Up to 74	90	67
	75+	44	33
**Acquired MRSA**		89	66
**Surgical procedure**		54	
	Clean	4	
	Clean-contaminated	32	
	Contaminated	18	
**Antibiotics use before diagnosis of MRSA**		66	
Penicillins		8	
Second-generation cephalosporins		28	
Third-generation cephalosporins		10	
Carbapenems		22	
Fluoroquinolones		12	
Aminoglycosides		2	
Others		2	
**Clinically significant MRSA infection**		69	51
**Type of infection**	Surgical site	23	
	Genitourinary	15	
	Blood stream	11	
	Gastrointestinal	11	
	Respiratory	9	
**Imported MRSA**		45	34

Of the 134 MRSA cases in the urology ward, 69 (51%) developed a CSMI. The major type of CSMI were surgical site (33%), genitourinary tract (22%) (Table 1). No patient died of CSMI. The incidence of CSMI decreased after the stepwise implementation of the strategies (coefficient for Year, -0.17; 95% CI, -0.25 to −0.087; *p* < 0.0001) (Figure 3C).

The incidence of MRSA in the urology ward increased transiently in 2007 when preoperative surveillance cultures of surgical sites was initiated, and decreased again thereafter. Figure 3D shows the submission rate of bacteriological cultures in the urology ward, which was calculated as a total number of submitted bacteriological cultures divided by total number of admissions during a year. Before initiating surveillance cultures, those were obtained in 20% of admitted patients in 2006 that were at risk for carrying MRSA. After introducing surveillance cultures, the submission rate of bacteriological cultures increased to 61% in 2008.

## Discussion

Our study demonstrates that a large-scale, long-term stepwise infection control strategy that includes the reduction or avoidance of antimicrobial prophylaxis in minimally invasive surgery contributed to a reduction in hospital-acquired MRSA and clinically significant MRSA infections in a urology ward, although the incidence of MRSA throughout the entire hospital increased despite the shared antimicrobial control policies [6]. Performing appropriate antibiotic stewardship, ensuring proper hand hygiene, cohorting the care of infected or colonized patients, decreasing unnecessary patient contact, and decreasing the length of stay for hospitalized patients are well-known strategies to prevent the nosocomial transmission of MRSA [28]. Despite the fact that continuous education of the hospital staff in regard to the transmission of resistant bacteria and the introduction of hospital-wide wall posters reminding individuals to keep their hands clean have been instituted throughout our hospital, the implementation of these measures has not resulted in a reduction in the incidence of MRSA. In addition to the aforementioned measures, in the urology ward, we introduced the reduction of AMP in gasless single-port surgery as a novel minimally invasive urologic surgery in 2001. Especially in clean urological surgeries, AMP has been avoided since 2005 without an increase in the number of SSIs [11]. After implementation of our strategy, the incidence of acquired MRSA decreased gradually despite the increase throughout the entire hospital. These findings indicate that a reduction or avoidance of AMP in minimally invasive urologic surgery could contribute to the management of MRSA in the urology ward. We believe that the minimally invasive surgery itself also was associated with the favorable outcomes. Technically, the rinsing of the operative field and subcutaneous space with sterile saline and subcuticular suture without additional dressing may also contribute to the reduction of SSIs, whose causative agent is often MRSA [29]. To the best of our knowledge, this is the first study in regard to the association between the incidence of MRSA and minimally invasive surgery.

In the urology ward, the incidence of MRSA increased transiently in 2007 and was followed by a decrease thereafter. We interpreted this phenomenon to be a result of the introduction of preoperative surveillance cultures of surgical sites in all patients undergoing surgery. This measure may lead to the detection of MRSA that had gone unrecognized during the preceding period. This is also supported by the increased proportion of patients with MRSA that were detected through surveillance cultures. Active surveillance to screen all patients for MRSA is more labor intensive and costly than passive surveillance in the routine course of patient care. McGinigle et al. reported that the detection of MRSA increased by 30 to 135% with active surveillance cultures and that what actually increased was the detection of patients colonized with MRSA [30].

The mechanism regarding the association between the controlled use of antibiotics and the incidence of MRSA has been discussed. The prolonged administration of antibiotic therapy or inadequate dosing of antibiotics may select for methicillin-resistant isolates in an overall population of *S. aureus*[31]. Several studies have suggested a positive association between antimicrobial consumption and the incidence of MRSA colonization or infection within the hospital setting [3,28]. Length of hospital stay has been demonstrated to be a predisposing factors in the acquisition of MRSA [32]. It is of note that, despite the fact that throughout the entire hospital the mean duration of hospital stay decreased by approximately 40%, the incidence of MRSA increased. The reason for this discrepancy remains unknown.

This study has limitations and the results must be interpreted with care. First, the study was conducted in a single center in Japan. Second, this study was not a case control study and it is possible that unmeasured confounders, such as the staff compliance to the hospital-wide standard precautions, the number of contaminated surgeries, underlying comorbidities as infectious risk and antibiotic consumption, were associated with the incidence of MRSA. Therefore, we could not clarify the impact of the reduction or avoidance of AMP and minimally invasive surgery on the incidence of MRSA because of the observational design.

## Conclusions

Our study demonstrates that a large-scale, long-term stepwise infection control strategy that includes the reduction or avoidance of antimicrobial prophylaxis in minimally invasive surgery over 10 years could contribute to the reduction in hospital-acquired MRSA and clinically significant infections in a urology ward of a university hospital.

## Abbreviations

MRSA: Methicillin-resistant *Staphylococcus aureus*; AMP: Antimicrobial prophylaxis; SSI: Surgical site infection; CSMI: Clinically significant MRSA infection; LVFX: Levofloxacin; TAZ/PIPC: Tazobactam sodium/piperacillin sodium; CI: Confidence interval.

## Competing interests

The authors declare that they have no competing interests.

## Authors’ contributions

KK has made substantial contributions to conception and design; MT, MI, SY, TK and MY have made acquisition of data, or analysis and interpretation of data; HM, KS, FK, SK and YF have been involved in drafting the manuscript or revising it critically for important intellectual content; and KK and HM have given final approval of the version to be published.

## Pre-publication history

The pre-publication history for this paper can be accessed here:

http://www.biomedcentral.com/1471-2490/13/35/prepub
